# Detection of Subclinical Diabetic Retinopathy by Fine Structure Analysis of Retinal Images

**DOI:** 10.1155/2019/5171965

**Published:** 2019-07-04

**Authors:** Maziyar M. Khansari, William D. O'Neill, Richard D. Penn, Norman P. Blair, Mahnaz Shahidi

**Affiliations:** ^1^Department of Ophthalmology, University of Southern California, Los Angeles, CA, USA; ^2^USC Stevens Neuroimaging and Informatics Institute, Keck School of Medicine of University of Southern California, Los Angeles, CA, USA; ^3^Department of Bioengineering, University of Illinois at Chicago, Chicago, IL, USA; ^4^Department of Neurosurgery, Rush University and Hospital, Chicago, IL, USA; ^5^Department of Ophthalmology and Visual Sciences, University of Illinois at Chicago, Chicago, IL, USA

## Abstract

**Background and Objective:**

Diabetic retinopathy (DR) is a major complication of diabetes and the leading cause of blindness among US working-age adults. Detection of subclinical DR is important for disease monitoring and prevention of damage to the retina before occurrence of vision loss. The purpose of this retrospective study is to describe an automated method for discrimination of subclinical DR using fine structure analysis of retinal images.

**Methods:**

Discrimination between nondiabetic control (NC; *N* = 16) and diabetic without clinical retinopathy (NDR; *N* = 17) subjects was performed using ordinary least squares regression and Fisher's linear discriminant analysis. A human observer also performed the discrimination by visual inspection of the images.

**Results:**

The discrimination rate for subclinical DR was 88% using the automated method and higher than the rate obtained by a human observer which was 45%.

**Conclusions:**

The method provides sensitive and rapid analysis of retinal images and could be useful in detecting subclinical DR.

## 1. Introduction

Diabetic retinopathy (DR) is a microvascular complication of diabetes and the leading cause of vision loss among working-age adults in the developed world [1, 2]. A population-based study has shown that DR prevalence after 15 years of diabetes is over 97% [3]. Although DR is a vision-threatening disease, its progression can be substantially controlled with early diagnosis, intensive glycemic management, and other systemic treatments [2–4]. However, detection of individuals in whom manifest DR is impending is an ongoing challenge to healthcare systems worldwide.

Microaneurysms and dot hemorrhages are the earliest signs of DR detectable by conventional clinical methods. More severe DR stages are characterized by the presence of hard exudates, neovascularization, capillary nonperfusion, fluid-filled spaces, and retinal detachment [5]. Advanced treatments, such as laser therapy, intravitreal steroid injections, intravitreal anti-VEGF agent injections, or vitreous surgery, are used to prevent vision impairment in progressed DR [6]. These treatments, however, carry risks [7], and hence, it would be advantageous to identify diabetes-related retinal abnormalities at an earlier subclinical stage for close monitoring and potential application of preventative treatment to delay development of vision-threatening DR.

Since retinal abnormalities in clinical DR are well documented, automated DR diagnosis using image analysis methods has been reported previously [8–10]. However, to the best of our knowledge, automated subclinical DR detection has not been reported, likely due to the fact that retinal abnormalities at this early stage are generally not directly discernable by visual inspection of fundus image. However, with the recent advent of optical coherence tomography angiography (OCTA), studies have shown alterations in capillary density, foveal avascular zone (FAZ) size, vessel diameters, tortuosity, branching angles, and the ratio of vessel length to diameter in subclinical DR [6, 11–14]. These alterations were detected based on statistical significance which does not necessarily translate to clinical significance and does not allow detection of abnormalities in individual cases [15]. Nevertheless, the confirmed presence of retinal vascular abnormalities in subclinical DR provides a rationale for developing image analysis techniques for automated subclinical DR detection which can be a potentially useful screening tool for the already large and rapidly growing diabetic population.

We showed in a previous study that fine structure analysis of conjunctival microvascular images can be useful for detecting clinical stages of DR [16], and this technique is highly sensitive to changes in vessel morphology such as tortuosity, vasodilation, and vasoconstriction [17]. The purpose of the current retrospective study is to test the hypothesis that fine structure analysis of retinal images can discriminate subclinical DR from nondiabetic controls.

## 2. Materials and Methods

### 2.1. Subjects

A total of 33 subjects (6 females and 27 males) participated in the study. Subjects underwent a comprehensive clinical retinal examination and were classified into nondiabetic control (NC; *N* = 16) and diabetic without clinical retinopathy (NDR; *N* = 17) subjects. The study was conducted in accordance with the tenets of the Declaration of Helsinki. An institutional review board of the University of Illinois at Chicago approved the current study. The study was explained to the subjects, and informed consents were obtained in accordance with the tenets of Declaration of Helsinki. Subjects' age (mean ± standard deviation (SD)) was 56 ± 9 years and 53 ± 10 years in NC and NDR subjects, respectively (*P*=0.2). The diabetes duration and hemoglobin A1C levels in NDR were 10 ± 8 years and 8 ± 2%, respectively. Three of the NDR subjects had coronary heart disease. The NDR and age-matched NC subjects were selected from our previous study [18], based on availability of good quality fundus images.

### 2.2. Image Acquisition and Processing

**Figure 1:**
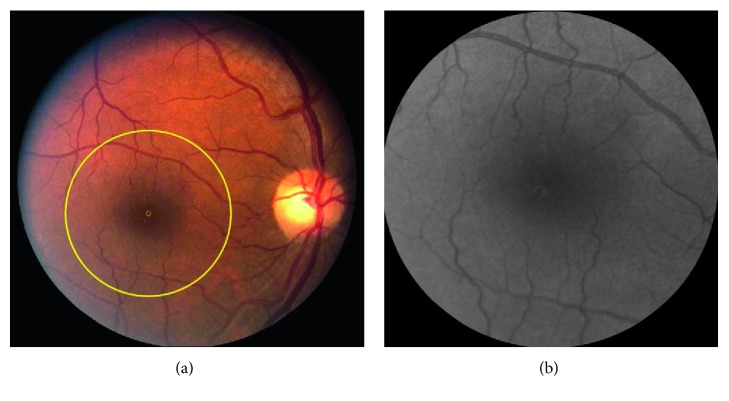
(a) Example of a color retinal image acquired in a nondiabetic control subject. A circular region of interest (ROI) with a diameter of 3.6 mm centered on the fovea and outlined by a yellow circle was selected for discrimination analysis. The small yellow circle in the center of the large circle shows the center of the fovea. (b) Converted grayscale images of the ROI in the same subject.

Imaging was performed by a commercially available fundus camera system with a 60° field of view. Images were acquired in color, and each one consisted of 2392 × 2048 pixels covering optic nerve head and the macula. A circular area of interest (ROI) with a radius of 3.6 mm (1000 pixels) centered on the fovea was selected from each image and converted to grayscale for analysis. Selection of this area allowed analysis of consistent regions between the subjects and was based on the assumption that retinal vascular alterations in subclinical DR are more likely to be detectable in smaller vessels and capillaries [6].  Figure 1 shows an example of selected ROI outlined by a yellow circle overlaid on the fundus images and converted to grayscale in a NC subject.

Fundus image discrimination was performed by a previously described fine structure image analysis method using a custom algorithm written in MATLAB (Release 2015b, MathWorks, Inc., Natick, MA, USA) [19]. In summary, pixels of each fundus ROI were shifted 1- or 2-pixel column-wise, row-wise, and along the diagonal to provide 8 different representations of the original images. Pixels in columns of the shifted images were stacked over each other to provide a 1D vector representation of the original image. The vectors formed a matrix and a column of ones was added to the foremost left column of this matrix to improve the discrimination by removing a parameter estimate bias due to nonzero sample means. A model image (*y*_*i*,*j*_) was defined as the weighted sum of the shifted images plus a zero-mean random process error term (*u*_*i*,*j*_) as shown in the following equation:

(1) $$\begin{array}{c} y_{i,j}=\overset{2}{\underset{k=0}{\sum }}\overset{2}{\underset{l=0}{\sum }}b_{k,l}y_{i-k\cdot j-l}+u_{i,j},\;k+l>0, \end{array}$$

where *b*_*k*,*l*_ are the coefficients needing estimation. Ordinary least square (OLS) regression was used to estimate the *b*_*k*,*l*_ parameters by minimizing the variance of error term. The estimated *b*_*k*,*l*_ covariance matrices for each class are the source of discrimination information.

For each group of subjects (i.e., NC and NDR), a representative matrix was generated where each column of the matrix was formed by *b*_*k*,*l*_ parameters that were obtained from each image in the group. Fisher's linear discriminant (FLD) analysis was used to determine a projection vector (*v*) based on the 2 matrices to project *b*_*k*,*l*_ parameters onto a scalar axis (*z*-projection). For 2 groups of images acquired from *N*_1_ and *N*_2_ subjects, 3 matrixes of *B*_1_, *B*_2_, and a pooled sample *B*_p_ were formed. *B*_1_ and *B*_2_ contained OLS coefficients for group 1 and group 2, respectively, and *B*_p_ was *B*_1_ stacked over *B*_2_. Finally, covariance matrices Ω_*s*_ of the *B*_*s*_, *s* = 1, 2, and *p*, were estimated and used to determine the projection vector *v* as shown in the FLD eigenvalue equation:

(2) $$\begin{array}{c} \left(n_{1}\Omega_{\text{p}}-n_{2}\Omega_{1}-n_{3}\Omega_{1}\right)v=\gamma_{1}\left(n_{2}\Omega_{1}+n_{3}\Omega_{2}\right)v, \end{array}$$

where *n*_1_ = *N*_1_ + *N*_1_ − 1, *n*_2_ = *N*_1_ − 1, and *n*_3_ = *N*_2_ − 1 and *γ*_1_ is the only nonzero eigenvalue. The projection vector *v* provides maximum absolute difference between the sample means of the 2 groups, while normalized by the sum of the covariance of each group.

**Figure 2:**
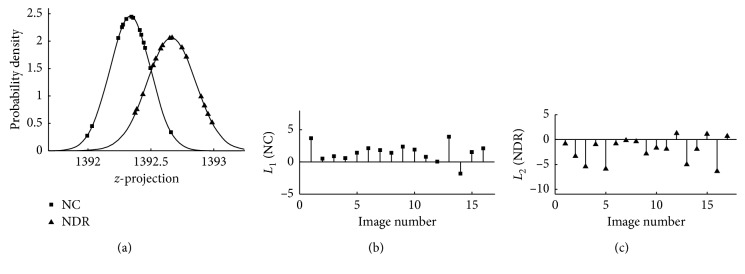
(a) Probability density of *z*-projections. (b) *L*_1_ and (c) *L*_2_ values between nondiabetic control (NC; group 1) and diabetic without retinopathy (NDR; group 2) subjects. Correctly classified images in group 1 had positive *L*_1_ values, while correctly classified images in group 2 had negative *L*_2_ values. The larger *L*_1_ value for an image in group 1 and the smaller *L*_2_ value for an image in group 2 are indicators of more likely true positive and more likely true negative discrimination, respectively.

Normality of distributions of *z*-projections was verified using Kolmogorov–Smirnov (KS) test to allow Kullback–Leibler discrimination (KLD) statistics as an indicator of likelihood of accurate discrimination. KLD of a discrimination function *L*(*z*) was calculated as shown in the following equation:

(3) $$\begin{array}{c} L_{1,2}\left(z\right)=\text{Ln}\left(\frac{f_{1}\left(z\right)}{f_{2}\left(z\right)}\right)=\text{Ln}\left(\frac{s_{2}}{s_{1}}\right)+\frac{{\left(z-m_{2}\right)}^{2}}{2s_{2}^{2}}-\frac{{\left(z-m_{1}\right)}^{2}}{2s_{1}^{2}}, \end{array}$$

where *f*_1_(*z*) and *f*_2_(*z*) are 2 *z*-projection density functions (Figure 2), *m*_1_ and *m*_2_ are sample means of the *z*-projection functions, and *s*_1_ and *s*_2_ are SD of the *z*-projection functions. When 2 groups of images are perfectly discriminated, *L*_1_ values for all the images in group 1 are positive and *L*_2_ values for all the images in group 2 are all negative. Consequently, misclassified images in group 1 and misclassified images in group 2 have negative and positive *L*_1_ and *L*_2_ values, respectively. Additionally, the higher *L*_1_ value for a group 1 image and the lower *L*_2_ value for a group 2 image indicate the higher likelihood of accurate discrimination. Discrimination rate was determined as the percentage ratio of the number of correctly discriminated images to the total number of the images in the 2 groups. Additional description of the fine structure technique may be found elsewhere [19].

### 2.3. Human Observer Image Discrimination

An experienced retinal specialist masked to subjects' diagnosis, and the result obtained by the automated discrimination, served as human observer and performed image discrimination. Each of the ROIs was visually inspected and assigned to one of the two groups. The discrimination rate for the human observer was calculated using the same formula as that used for the automated method.

## 3. Results

The KS test results showed that the distribution of *z*-projects in NC and NDR subjects was normal (*P* < 0.001). The automated discrimination rate was 88% (29/33) with 1 and 3 misclassifications in NC and NDR subjects, respectively. The KLD statistics between the NC and NDR subjects are shown in Figure 2. The range of *L*_1_ values for correctly classified images in group 1 was between 0.1 and 4, while the range of *L*_2_ values for correctly classified images in group 2 was between −0.2 and −6. Discrimination rate by the human observer between NC and NDR subjects was 45%.

## 4. Discussion and Conclusion

In the current study, an automated fine structure discrimination [19] was performed for the first time for analysis of retinal images to detect subclinical DR. Additionally, the likelihood of accurate discrimination for each of the images in the 2 groups of subclinical DR and nondiabetic control was reported.

The rate of subclinical DR discrimination using the automated technique was higher than the rate obtained by the human observer, suggesting that the method can detect retinal alterations which cannot be visually discerned by a trained observer. DR diagnosis and progression monitoring are currently based on presence of vascular pathologies, such as microaneurysms and vascular leakage. Detection of abnormalities in this early stage may prompt assessment to optimize glycemic control and possibly add new treatment to prevent or delay DR progression. It can also be used as a basis to urge patients to optimize their diabetic control. Moreover, these early-stage alterations may suggest presence of undetectable microvascular alterations in other critical organs such as the kidney or brain. In fact, association between diabetes and presence of microvascular alterations in various tissues such as brain, nail fold, and conjunctiva have been reported previously [18, 20–25]. Therefore, the method shows promise to improve monitoring and managing diabetic-related disorders throughout the body.

DR is a progressive complication of diabetes that causes vision impairment by affecting retinal vessels that supply inner retinal layers [26]. Alterations in intercapillary area, capillary density, and FAZ size were shown to be correlated with the progression of DR [6, 27, 28]. Furthermore, retinal imaging by an adaptive optics confocal scanning laser ophthalmoscope [29] showed a significant increase in tortuosity of retinal arteriovenous channels at subclinical DR [6]. Furthermore, changes in retinal oxygenation, resistive index, and blood flow have been reported in subclinical DR [27, 28, 30]. These and other retinal physiological alterations can cause vasodilation, vessel wall stiffening, and tortuosity alterations in subclinical DR which may not be visually detected by clinical evaluations. However, techniques such as fine structure analysis which use all the information in the image rather than specific microvasculopathies might be able to provide automated subclinical DR detection.

At subclinical DR stage, there are previously reported changes in the retinal vasculature, including alterations in tortuosity of retinal arteriovenous channels, retinal oxygenation, resistive index, blood flow, vessel caliber, and vessel wall stiffness [6, 26–30]. It is likely that the fine structure analysis of the retinal images detects such pathologies as a basis for discrimination. Detection of global alterations by fine structure is based on statistical and mathematical analysis at pixel level. Hence, each of the 3 × 10^6^ ROI pixels is treated as a feature that can influence the discrimination result. This provides detection of global alterations such as vascular integrity and their pattern at an early stage, prior to visualization by clinical examination in which gross vascular changes are detectable. However, further studies are needed to determine which of these changes have greater influence on the fine structure analysis.

We believe that shortly after diabetes, abnormalities begin to develop and reach a threshold over years manifested by DR. The fine structure analysis is highly sensitive to these premature abnormalities and hence provides early diagnosis on the course of the disease. Moreover, the KLD statistics provide quantitative representation of severity of abnormalities rather than only on an ordinal scale. Furthermore, the algorithm has the advantage of requiring short computational time (e.g., less than 5 seconds on a 1.3 GHz system with 8 GB RAM) which offers substantial potential for its application to very large image sets. In fact, the method may have a significant clinical impact for subclinical DR detection, particularly due to the expected increase in the prevalence of diabetes and shortage in the number of qualified screening healthcare providers [31].

In the current study, the macular region selected for analysis contains mostly smaller caliber vessels that are more vulnerable to the disease than the larger ones [6]. Selection of this region also allowed analysis of a consistent area among the subjects. In future, inclusion of different size retinal regions in the analysis can be useful for determining the effect of regional differences on the discrimination rates. Also, the specificity of the current technique was not determined in the current study and future research is needed to determine whether it can discriminate between DR and other retinal diseases. It is important to note that since it takes time for diabetes-related abnormalities to develop [32], it is expected that there will be an interval between the onset of diabetes and the development of abnormal test results using the fine structure method. Hence, future studies are needed to determine the performance of the automated discrimination with changes over time and investigate potential correlation between the KLD values and duration of diabetes. Such a study can also demonstrate whether the glycemic treatment can reverse the level of abnormalities detected by the automated method. Finally, future studies are needed to determine the clinical utility of the current technique in evaluating, monitoring, and treating subclinical DR. Nevertheless, the finding of the current study demonstrates the potential for fine structure analysis to detect subclinical DR based on retinal images.

## Data Availability

The data used in this study are not publicly available due to institutional restrictions.
